# An ABCC8 Nonsense Mutation Causing Neonatal Diabetes Through Altered Transcript Expression

**DOI:** 10.4274/jcrpe.4624

**Published:** 2017-09-01

**Authors:** Sarah E. Flanagan, Vũ Chí Dũng, Jayne A. L. Houghton, Elisa De Franco, Can Thi Bich Ngoc, Annet Damhuis, Frances M. Ashcroft, Lorna W. Harries, Sian Ellard

**Affiliations:** 1 University of Exeter Medical School, Institute of Biomedical and Clinical Science, Department of Molecular Genetics, Exeter, United Kingdom; 2 National Children’s Hospital, Department of Endocrinology, Metabolism and Genetics, Hanoi, Vietnam; 3 University of Oxford, Henry Wellcome Centre for Gene Function, Department of Physiology, Anatomy and Genetics, Oxford, United Kingdom

**Keywords:** neonatal diabetes, nonsense mutation, splicing

## Abstract

The pancreatic ATP-sensitive K^+^ (K-ATP) channel is a key regulator of insulin secretion. Gain-of-function mutations in the genes encoding the Kir6.2 (KCNJ11) and SUR1 (ABCC8) subunits of the channel cause neonatal diabetes, whilst loss-of-function mutations in these genes result in congenital hyperinsulinism. We report two patients with neonatal diabetes in whom we unexpectedly identified recessively inherited loss-of-function mutations. The aim of this study was to investigate how a homozygous nonsense mutation in ABCC8 could result in neonatal diabetes. The ABCC8 p.Glu747* was identified in two unrelated Vietnamese patients. This mutation is located within the in-frame exon 17 and RNA studies confirmed (a) the absence of full length SUR1 mRNA and (b) the presence of the alternatively spliced transcript lacking exon 17. Successful transfer of both patients to sulphonylurea treatment suggests that the altered transcript expression enhances the sensitivity of the K-ATP channel to Mg-ADP/ATP. This is the first report of an ABCC8 nonsense mutation causing a gain-of-channel function and these findings extend the spectrum of K-ATP channel mutations observed in patients with neonatal diabetes.

**What is already known on this topic?**

The pancreatic ATP-sensitive K^+^ (K-ATP) channel regulates insulin secretion. Gain-of-function mutations in the genes encoding the two subunits of the channel, KCNJ11 and ABCC8, cause neonatal diabetes, whilst loss-of-function mutations in these genes result in congenital hyperinsulinism.

**What this study adds?**

This is the first report of a loss-of-function mutation in ABCC8 resulting in gain-of-channel function and neonatal diabetes.

## INTRODUCTION

The pancreatic beta cell K-ATP channel is crucial for the controlled release of insulin as evidenced by the identification of KCNJ11 and ABCC8 mutations in patients with neonatal diabetes and congenital hyperinsulinism (1). Loss-of-function mutations in these genes cause hyperinsulinism by leading to a loss of K-ATP channels at the plasma membrane via effects on gene expression, protein synthesis, protein maturation, or membrane trafficking or by impairing the ability of SUR1 to regulate channel activity (2,3). This latter effect is brought about by reducing or abolishing channel activation by MgADP and/or MgATP. In contrast, gain-of-function mutations cause neonatal diabetes by reducing the ability of ATP to inhibit channel activity (at Kir6.2) or enhancing the ability of Mg-nucleotides to stimulate channel activity (at SUR1) (4).

ATP-sensitive potassium (K-ATP) channels are hetero-octomeric complexes consisting of four pore-forming K^+^ inward rectifying (K_IR_6.x) subunits and four regulatory sulphonylurea receptor (SURx) subunits. The K_IR_6.x protein has a pore loop flanked by two transmembrane and cytoplasmic domains, whilst SURx consists of 17 transmembrane helices with 2 regulatory nucleotide binding domains (NBD). Binding of ATP to Kir6.x inhibits channel activity, whilst interaction of cytosolic Mg-nucleotides with SURx causes channel activation (5).

Different isoforms and splice variants of K-ATP channel subunits exist with different combinations of K_IR_6.x and SURx acting to increase channel diversity. The K-ATP channel within the pancreatic beta cell is composed of four Kir6.2 and four SUR1 subunits. Kir6.2 is encoded by the single exon of the KCNJ11 gene whilst ABCC8, which encodes SUR1, has 39 exons. At least five different SUR1 alternatively spliced transcripts have been identified in rodent cell lines which result from exon skipping (SUR1Δ17, Δ19, Δ17/19, Δ33) or truncation of the C-terminus (SUR1C) (6,7).

Recessively inherited K-ATP channel mutations are most common in patients with congenital hyperinsulinism, whilst dominant mutations are the commonest cause of neonatal diabetes. In a few patients with neonatal diabetes, recessive inheritance of two ABCC8 or KCNJ11 gain-of-function mutations or compound heterozygosity for an activating and an inactivating ABCC8 mutation have been described (8,9). We now report two patients with diabetes diagnosed before 6 months of age: one with homozygosity for a novel ABCC8 nonsense variant and one with compound heterozygosity for the same nonsense variant and a previously reported loss-of-function missense mutation.

## METHODS

For the genetic analysis of ABCC8 and KCNJ11 genes, genomic DNA was extracted from peripheral leukocytes using standard procedures and the coding regions and intron/exon boundaries of the ABCC8 and KCNJ11 genes were amplified by polymerase chain reaction (PCR) (primers available on request). Amplicons were sequenced using the Big Dye Terminator Cycler Sequencing Kit v3.1 (Applied Biosystems, Warrington, UK) according to manufacturer’s instructions and reactions were analysed on an ABI3730 Capillary sequencer (Applied Biosystems, Warrington, UK). Sequences were compared with the reference sequences (NM_000525.3 and NM_000352.3) using Mutation Surveyor v3.24 software (SoftGenetics, State College, PA).

Clinical information was provided by the referring clinicians via a neonatal diabetes request form (available at www.diabetesgenes.org) or by provision of clinical notes. The study was conducted in accordance with the Declaration of Helsinki principles with informed parental consent given on behalf of the children.

### Real-time Quantification of ABCC8 Transcripts with and without Exon 17

Total pancreatic RNA was purchased from Clontech (Oxford, UK), islets were sourced from the National Disease Resource Interchange (NDRI; Philadelphia, USA). These samples were taken from donors who were otherwise free of disease. Control samples were free of cancer, pancreatic fibrosis or systematic sepsis. Samples must have had a maximum of 5 mins ‘downtime’ before sample processing, and donors must have had no abdominal injuries or pancreatic trauma and no transmissible diseases. RNA was extracted using the Eppendorf Perfect RNA mini Kit (Eppendorf, Hamburg, Germany). 500 ng total RNA from each sample were reverse transcribed using the Thermoscript real-time-PCR (RT-PCR) system (Invitrogen, Paisley, UK) using 50 °C as the incubation temperature as per manufacturer’s instructions. TaqMan qRTPCR probes specific to the exon16:exon17 boundary or the exon16:exon18 were purchased from Life Technologies (Warrington, UK) and validated by standard curve analysis. Assay details are available on request. RT-PCR reactions were carried out using the ABI Prism 7000 platform (Applied Biosystems). Each sample was amplified in triplicate to ensure accuracy of quantification. Where multiple samples per tissue were tested, each sample was from a separate mRNA extraction and reverse transcription. PCRs contained 10 μL TaqMan Universal Mastermix (no AMPerase) (Applied Biosystems), 0.9 μmol/L each primer, 0.25 μmol/L probe, and 2 μL cDNA reverse transcribed as above in a total volume of 20 μL. PCR conditions were a single cycle of 95 °C for 10 min followed by 40 cycles of 95 °C for 15 s and 60 °C for 1 min. Transcript abundance for the full length and exon 17 deleted mRNAs were calculated using the Comparative Ct approach, relative to the endogenous control beta 2 microglobulin (B2M) and normalised to the level of full length ABCC8 transcript in each sample.

## RESULTS

The first patient is a male who presented with hyperglycaemia (blood glucose: 30.9 mmol/L [normal range 4-6 mmol/L (556 mg/dL)] on the 36^th^ day of life. At the age of 4 years, he was referred for genetic testing and at this time was treated with 0.7 U/kg/day of insulin and had a HbA1c of 8% (normal range <6.5%). Sequence analysis identified a novel homozygous nonsense variant, p.Glu747* (c.2239G>T), in exon 17 of ABCC8. No KCNJ11 mutation was identified and testing of the unaffected parents confirmed that both were heterozygous for the p.Glu747* variant.

Patient 2 is a male who presented with a blood glucose level of 26.5 mmol/L [normal range 4-6 mmol/L (477 mg/dL)] during the 6^th^ week of life. At the time of genetic testing, he was being treated with 1.2 U/kg/day of insulin and had a HbA1c of 10.3%. Sequence analysis identified two heterozygous ABCC8 variants: p.Glu747* (c.2239G>T) in exon 17 and p.Glu128Lys (c.382G>A) in exon 3. The p.Glu128Lys variant has been reported previously as a recessively acting loss-of-function mutation in multiple unrelated patients with congenital hyperinsulinism (10,11,12). No KCNJ11 mutations were identified and testing of the unaffected parents confirmed that the two variants were in trans with p.Glu128Lys inherited from the mother and p.Glu747* inherited from the father. A summary of the clinical characteristics and genetic results for these two unrelated patients are provided in Table 1.

**Figure 1:**
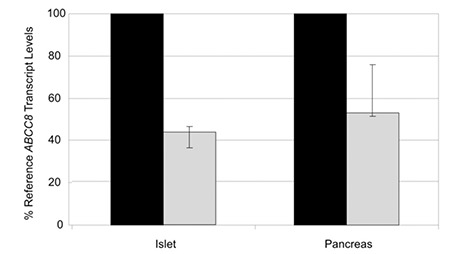
Expression pattern of ABCC8 transcripts with and without exon 17 in human pancreas and islets. The graphs illustrate the levels of messenger RNA transcripts either containing exon 17 (black bars) or excluding exon 17 (grey bars) expressed relative to levels of the full length ABCC8 reference transcript in human islets (3 technical replicates from RNA pooled from 2 independent donors) and from human total pancreas (3 technical replicates from RNA pooled from 3 donors). Transcript levels were determined by isoform-specific qRTPCR, calculated relative to the endogenous control beta 2 microglobulin (B2M) and normalised to the amount of full length transcript present in each sample. Error bars represent the range of quantification for each sample

**Figure 2:**
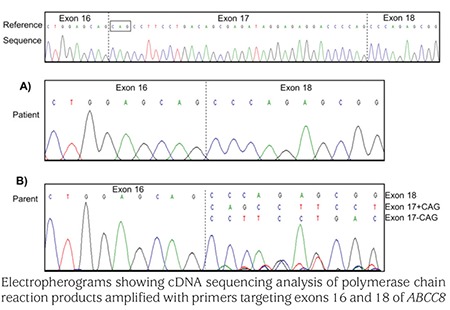
The reference genomic DNA sequence is provided. A dashed line denotes an exon-exon boundary. (A) Sequence analysis identified homozygosity for the SUR1Δ17 in patient 1 with the homozygous p.Glu747* ABCC8 variant in exon 17. (B) Sequence analysis of cDNA from the unaffected father who is heterozygous for the p.Glu747* variant identified a transcript which lacked exon 17 and two transcripts which contained exon 17. An alternate splice recognition site at the 3’ intron 16/exon 17 boundary results in two transcripts containing either 36 or 39 basepairs (+/- CAG boxed) (GenBank L78208 and L78224)

The identification of biallelic ABCC8 loss-of-function variants in patients with neonatal diabetes was unexpected as recessive loss-of-function K-ATP channel mutations usually cause congenital hyperinsulinism. The ABCC8 p.Glu747* nonsense variant is located within the small (36 bp) in-frame exon 17 that encodes part of the nucleotide binding domain 1 and there is a rodent SUR1 isoform which lacks exon 17 (6). We therefore undertook mRNA studies to investigate (a) the relative proportion of messenger RNA transcripts with and without this exon in control samples and (b) the effect of the p.Glu747* variant. We found that the isoform lacking exon 17 was expressed at only 44% of the level of the reference ABCC8 transcript in human islets and at 53% of levels of the reference ABCC8 transcript in whole pancreas (Figure 1). We next isolated total RNA from patient-derived leukocyte cell lines and amplified cDNA using PCR primers targeted to exons 16 and 18 (primer sequences available on request). Sequence analysis showed complete absence of transcripts containing exon 17 in the patient who was homozygous for the p.Glu747* variant. His father, who is heterozygous for the variant, was shown to have a mixture of transcripts with or without exon 17 (Figure 2).

Sulphonylureas are an effective treatment for individuals with neonatal diabetes caused by mutations in ABCC8 or KCNJ11 and result in improved glycaemic control (13,14). These drugs exert their effects by binding to the SUR1 subunit of the K-ATP channel and closing the channel independently of ATP. Studies undertaken by Hambrock et al (6) demonstrated high-affinity binding of sulphonylureas to the rat SUR1Δ17 isoform suggesting that sulphonylurea treatment may be effective in our two patients. We therefore undertook a controlled trial of glibenclamide in each patient which resulted in an improvement in glycaemic control (8.3% on insulin vs. 5.2% on glibenclamide in the first patient and 13.6% vs. 5.2% in the second). The patients are currently 11 years and 6 years and are well-controlled on 0.43 mg/kg/day and 0.24 mg/kg/day of glibenclamide, respectively. As sulphonlyureas bind directly to the SUR1 subunit, this observation confirms that the p.Glu747* variant is not preventing expression or trafficking of K-ATP channels to the cell surface. Patient 2 was compound heterozygous for the p.Glu747* variant and a p.Glu128Lys mutation. Functional studies have shown that p.Glu128Lys results in reduced expression of mutant channels (11). It is therefore likely that in both of our patients, all K-ATP channels at the cell surface are homomeric for the SUR1Δ17 isoform.

## DISCUSSION

To date, all reported gain-of-function KCNJ11 and ABCC8 mutations cause neonatal diabetes by altering the sensitivity of the K-ATP channel to intracellular MgADP or ATP. As SUR1 exon 17 encodes part of NBD1 which is required for nucelotide handling, it seems likely that a protein lacking this crucial regulatory domain would have an altered sensitivity to ATP. However, whilst an SUR2 splice variant lacking exon 17 has been identified in mice which had a 2-fold reduction in sensitivity to ATP compared to the full-length isoform (15), a study using rat SUR1Δ17 found no differences in sensitivity to MgATP between the mutant and wild-type channels (6). The reason for this discrepancy is not clear but may reflect differences between rodent and human K-ATP channel physiology. Therefore, whilst further studies are required to assess the effect of the p.Glu747* variant upon channel activity in humans, the identification of the same variant in two patients with sulphonylurea-responsive neonatal diabetes, and its location within an alternatively spliced exon encoding a functionally important domain, provides strong evidence that p.Glu747* is an etiological mutation.

In conclusion, we have identified a novel recessively-inherited ABCC8 nonsense variant in two unrelated patients with neonatal diabetes. We hypothesize that this variant causes a gain-of-channel function by changing the relative abundance of alternatively spliced SUR1 transcripts which enhances the sensitivity of the channel to intracellular MgADP/ATP. These results expand knowledge on genotype/phenotype relationships in neonatal diabetes due to K-ATP channel mutations and highlight the importance of considering the impact on different transcripts when unexpected mutations are identified in genes that are alternatively spliced.

## Figures and Tables

**Table 1:**
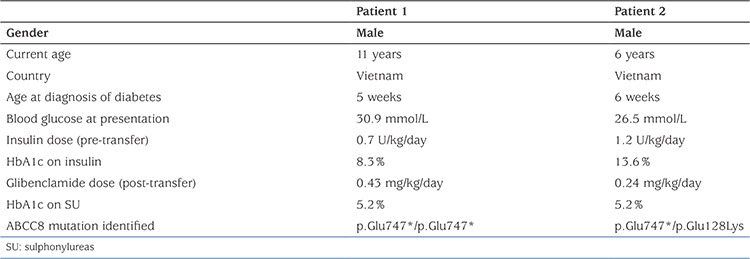
Clinical characteristics and results of genetic analysis in the two patients
